# Involvement of DPP3 in modulating oncological features and oxidative stress response in esophageal squamous cell carcinoma

**DOI:** 10.1042/BSR20222472

**Published:** 2023-09-06

**Authors:** Mohit Arora, Sarita Kumari, Lokesh Kadian, Geethadevi Anupa, Jay Singh, Anurag Kumar, Deepika Verma, Raja Pramanik, Sunil Kumar, Rajni Yadav, Anita Chopra, Shyam S. Chauhan

**Affiliations:** 1 Department of Biochemistry, All India Institute of Medical Sciences, New Delhi, India; 2 Laboratory Oncology Unit, Dr. BRA-IRCH, All India Institute of Medical Sciences, New Delhi, India; 3 Department of Medical Oncology, All India Institute of Medical Sciences, New Delhi, India; 4 Department of Surgical Oncology, All India Institute of Medical Sciences, New Delhi, India; 5 Department of Pathology, All India Institute of Medical Sciences, New Delhi, India

**Keywords:** Dipeptidyl peptidase, esophageal cancer, esophageal squamous cell carcinoma, Nrf2, oxidative stress

## Abstract

Resistance to therapy in esophageal squamous cell carcinoma (ESCC) is a critical clinical problem and identification of novel therapeutic targets is highly warranted. Dipeptidyl peptidase III (DPP3) is a zinc-dependent aminopeptidase and functions in the terminal stages of the protein turnover. Several studies have reported overexpression and oncogenic functions of DPP3 in numerous malignancies. The present study aimed to determine the expression pattern and functional role of DPP3 in ESCC. DPP3 expression was assessed in normal and tumor tissues using quantitative real-time (qRT)-PCR and corroborated with ESCC gene expression datasets from Gene Expression Omnibus (GEO) and The cancer genome atlas (TCGA). DPP3 stable knockdown was performed in ESCC cells by shRNA and its effect on cell proliferation, migration, cell cycle, apoptosis, and activation of nuclear factor erythroid 2-related factor 2 (NRF2) pathway was assessed. The results suggested that DPP3 is overexpressed in ESCC and its knockdown leads to reduced proliferation, increased apoptosis, and inhibited migration of ESCC cells. Additionally, DPP3 knockdown leads to down-regulation of the NRF2 pathway proteins, such as NRF2, G6PD, and NQO1 along with increased sensitivity toward oxidative stress-induced cell death and chemotherapy. Conclusively, these results demonstrate critical role of DPP3 in ESCC and DPP3/NRF2 axis may serve as an attractive therapeutic target against chemoresistance in this malignancy.

## Introduction

Esophageal cancer (EC) is the sixth leading cause of cancer-related deaths worldwide [1]. Esophageal squamous cell carcinoma (ESCC) accounts for more than 80% of EC cases in Asia [2,3]. Therapy resistance remains a major hurdle in the management of EC and many patients suffer severe side effects from the neoadjuvant chemo-radiotherapy with minimal clinical benefit [4,5]. In addition, many patients who initially respond to therapy may suffer a relapse within 3 years, conferring 5-year survival of only 20% [4]. Clearly, there is a need to identify molecular features which can predict or modulate treatment response, so that alternative strategies can be planned.

Most chemotherapies partially exert their cytotoxic roles by induction of oxidative stress in cells [6–8]. Response to oxidative stress in normal cells is mediated by nuclear factor erythroid 2-related factor 2 (NRF2) signaling [9]. Under unstressed conditions, low cellular levels of NRF2 protein in the cytoplasm are maintained by its KEAP1-dependent ubiquitination followed by degradation. Upon oxidative stress, ubiquitination of NRF2 is blocked, leading to the stabilization and nuclear translocation of NRF2 and subsequent induction of its target genes including Glucose-6-phosphate dehydrogenase, microsomal glutathione S-transferase 1, NAD(P)H dehydrogenase [quinone] 1, thioredoxin 1, glutathione synthetase, etc., which are involved in redox balance, purine metabolism, lipid metabolism, inflammation, and proteostasis [9]. Overexpression of the NRF2 pathway has been widely recognized to be associated with drug resistance in various malignancies, including EC [10,11]. Therefore, inhibition of this pathway has been assessed as therapeutics [11,12]. A common mechanism of NRF2 overactivation in malignancies involves mutations in NFE2L2 (gene that encodes for NRF2), KEAP1, and CUL3 genes [13,14]. Commonly, these mutations inhibit the interaction between NRF2 and KEAP1 or the ubiquitination of NRF2. NRF2 pathway genes have been observed to be mutated in up to 25% of ESCC patients [15–17], which leads to overactivation of this signaling pathway. However, immunohistochemical staining revealed the predominance of aberrant NRF2 signaling in 48.68% of ESCC, much higher than this pathway's mutation rates reported in previous studies, indicating the involvement of other factors in NRF2 activation [10]. Consistent with this observation, at least five additional mechanisms are known to activate NRF2 in cancer: hypomethylation of KEAP1, accumulation of disruptor proteins, increased production of NRF2, electrophoretic attack of KEAP1 by oncometabolites, and down-regulation of NRF2-targeting microRNAs (miRNAs) [18]. Several disruptor proteins such as DPP3, p62, and PALB2 compete with Nrf2 to bind Keap1, resulting in Nrf2 overactivation [18].

Dipeptidyl peptidase III (DPP3; EC 3.4.14.4) is a cytosolic aminopeptidase belonging to the M49 peptidase family [19]. It is ubiquitously expressed in the human body and acts in the terminal stages of protein turnover [19,20]. It is shown that DPP3 activity is altered in various pathophysiological conditions such as cardiogenic shock, sepsis, and heart failure [19,21]. Importantly, overexpression and aberrant activity of DPP3 have been reported in several malignancies, including ovarian cancer, breast cancer, lung cancer, and cervical cancer [22–24]. Apart from its peptidase activity, DPP3 is involved in the regulation of the NRF2 pathway by having an ETGE motif to interact with KEAP1 [25]. In breast cancer, it was shown that DPP3 expression during oxidative stress induction leads to up-regulation of the NRF2 pathway [20]. The clinical significance of this peptidase in cancers has been recently highlighted by its membership as a cancer signature gene in a six-gene model, which has been demonstrated to have diagnostic and prognostic value in breast and lung cancers [26]. Accumulating evidence regarding the important role of DPP3 in pathophysiology has led to a recent surge of interest in the biology of this peptidase [27–32]. Most recently, it was shown that circulating DPP3 levels may act as a biomarker for acute medical conditions such as heart and kidney failure or septic shock [33,34]. In the present study, the expression pattern and the molecular functions of DPP3 in ESCC were investigated. It was also determined whether DPP3 mRNA levels or serum levels in ESCC can be utilized as prognosticator in this disease. Additionally, the effect of DPP3 knockdown on cellular functions and NRF2 signaling in EC cells were determined.

## Methods

### Patients and tissue samples

Tissue samples and/or peripheral blood were collected from patients who reported at the institutional cancer clinic, Dr. B.R.A-IRCH, AIIMS, New Delhi, India for endoscopic biopsy due to suspicion of having EC. A biopsy tissue from the lesion suspected of being a tumor was collected along with a biopsy from adjacent normal tissue in RNAlater solution (Sigma-Aldrich, U.S.A.) and stored at −80°C. Additionally, 5 ml of peripheral blood was collected before biopsy in an EDTA vial and processed for serum isolation. After confirmation of the diagnosis of a tumor from a biopsy of the same lesion, ESCC tissue along with adjacent normal tissue was processed for RNA extraction and gene expression analysis. A total of 70 patients were included in the study. ESCC and esophageal adenocarcinoma (EAD) were diagnosed in 56 and 9 patients, respectively. As some of the patients could provide consent only for tissue or peripheral blood, normal-tumor paired tissues were available from 41 patients, among which 32 were ESCC and 9 were EAD. Serum was isolated from the peripheral blood of 47 ESCC patients (mean age: 52.35 years), 8 EAD patients (mean age: 52.24 years), and from 22 healthy controls (mean age: 39.31 years). Exclusion criteria for patients were age <18 years, other active malignancy or history of malignancy, and the presence of metastatic disease. Exclusion criteria for healthy control were age <25 years, history of other malignancy or recent history of any chronic inflammatory disease, ongoing pregnancy, or breastfeeding. The study was approved by the institute’s ethics committee and informed consent was obtained from all patients and healthy volunteers following the Declaration of Helsinki.

### Enzyme-linked immunoassay

Sandwich enzyme-linked immunosorbent assay was used to determine the serum levels of DPP3 using the commercially available kit (#3019344, RealGene Labs, CA, U.S.A.) as per the manufacturer’s recommended protocol. Briefly, a 96-well plate precoated with a monoclonal antibody that was specific to DPP3 was utilized. The wells were filled with standards of known concentrations given in the kit and serum (test) samples, and they were left at room temperature for 2.5 h. After incubation, the wells were washed four times with wash buffer, aspirating and decanting the solution onto fresh paper towels each time. This was followed by incubation with the biotinylated DPP3-specific antibody for 1 h. Washing was repeated four times after which avidin-conjugated horseradish peroxidase solution was added and incubated for 45 min at room temperature. This was followed by washing and adding TMB substrate solution to develop color. The reaction was terminated by the addition of sulfuric acid solution and the amount of color produced was measured at 450 nm with a 96-well plate reader. The amount of test antigen was then calculated based on the standard curve plotted using Graphpad Prism version 8.

### Collection and analysis of cancer genomics data

Gene expression data from microarray or RNA-sequencing studies of ESCC were retrieved from Gene Expression Omnibus (GEO) website (https://www.ncbi.nlm.nih.gov/gds) and The Cancer Genome Atlas (TCGA) datasets. Normal esophageal tissue RNA-sequencing data from GTEx and tumor tissue RNA-sequencing data from TCGA-ESCA study were assessed as transcript per million (RSEM-TPM) values from the UCSC Xena browser (https://xena.ucsc.edu/) using GTEx-TCGA-TARGET combined study data. Expression of DPP3 and NRF2 pathway genes in four GEO datasets GSE23400, GSE75241, GSE20347, and GSE70409 contained gene expression data of paired normal esophageal and ESCC tissues. Data from GSE29001 utilized laser capture microdissection to isolate normal esophageal basal layer, normal esophageal differentiated squamous epithelia, and esophageal tumor area for gene expression. Copy number data of TCGA EC study were assessed from cBioPortal [35,36]. To determine the relative expression of DPP3 in different EC cell lines, we assessed Cancer Cell Line Encyclopedia (CCLE) cell line study datasets through cBioPortal.

### Survival analysis

We assessed the association of DPP3 mRNA expression with patient survival in the TCGA EC dataset where information for four survival parameters, i.e., overall survival (OS), disease-specific survival (DSS), disease-free survival (DFS), and progression-free survival (PFS) was available for 94 ESCC patients. To differentiate patients into two groups based on DPP3 expression, we used a cutoff value of standard deviation of 1.5 above the mean (Z score >1.5) expression to be grouped as patients with high DPP3 expression consisting of 14 patients, while the remaining 80 patients were classified as DPP3 low-expression group. To determine the association of DPP3 copy number with patient survival, the DPP3 copy number status of amplification and gain was used to make two groups of patients; the first group consisted of patients with either DPP3 gain or amplification, and another group of remaining patients, which may have other copy number alteration. Log-rank *P-*values were retrieved with a maximum follow-up of 65 months.

### Cell line culture and chemicals

ESCC cell lines KYSE-410 and TE-10 were procured from Sigma-Aldrich (Bangalore, India) and ATCC (Rockville, Maryland, MD, USA). Cell lines were maintained under RPMI media (#SH30027.01, Hyclone, U.S.A.) with 10% FBS and antibiotics (penicillin, streptomycin, and amphotericin B; 15240-062, ThermoFisher Scienctific Inc., U.S.A.). Cells were incubated at 37°C, 5% CO_2_ overnight. HEK-293T cell lines were cultured for lentivirus production in Dulbecco’s Modified Eagle Medium (DMEM) media (BI, Cromwell, CT, U.S.A.) with 10% FBS and antibiotics. For chemosensitivity experiments, both cell lines were treated with drugs cisplatin, 5-flourourecils, carboplatin, and paclitaxel (Cayman Chemicals, U.S.A.) maintained in 5% FBS and antibiotics.

### Lentiviral production and transfection in KYSE-410

For lentivirus production, shRNA for targeting DPP3 was cloned in pLKO.1 plasmid along with psPAX2 packaging plasmid and pMD2.G envelope plasmid. The shRNA plasmid, packaging plasmid, and envelope plasmid were mixed in Opti-MEM media (Gibco™, Thermo Fisher Scientific, U.S.A.) and incubated with FuGENE® HD Transfection Reagent at room temperature for 20 min. This mix was added dropwise to HEK-293T cells cultured 24 h before transfection in antibiotic-free DMEM media (Sigma-Aldrich, U.S.A.). After 48 h, media containing the virus were collected and filtered with a 0.45-μM filter. For infection using, shRNA and control plasmid, 1 ml virus and 10 µg/ml polybrene were added to 5 × 10^5^ KYSE-410 cells in a six-well plate. The plate was spun at 931 ***g*** for 90 min at 30°C. Media were removed and 2 ml of fresh media without polybrene was added dropwise to each well. After 48 h, the media were replaced with selection media containing 0.75 μg/ml puromycin. Media were changed after every 2 days with fresh puromycin-containing media for selection up to 14 days followed by validation of knockdown efficiency.

### Quantitative real-time PCR

RNA was extracted using TRIzol (Thermo Fisher Scientific, Waltham, MA, U.S.A.) reagent using the manufacturer’s instructions. The quality and quantity of RNA were assessed by a Nano volume spectrophotometer (Thermo Fisher Scientific, U.S.A.). RNA was reverse transcribed to cDNA using random hexamers, RNase inhibitors, dNTPs, and M-MuLV reverse transcriptase enzyme (Fermentas, U.S.A.). The expression levels of the analyzed gene were measured by real-time PCR (CFX96™, Bio-Rad, U.S.A.) using SsoFast EvaGreen supermix (Bio-Rad, U.S.A.). The respective primer sequences of analyzed genes are given in Table 1. In all cases, samples were run in triplicates. Ct values were normalized with 18S as the housekeeping gene.

**Table 1:** List of primers for quantitative real-time-PCR analysis

Gene	Forward primer sequence	Reverse primer sequence
18S	GTAACCCGTTGAACCCCATT	CCATCCAATCGGTAGTAGCG
DPP3	TGGACTCACAGAACCTCAGTGC	AGCTTGGAAGTCACCTCAGAGT
NRF2	CAGCGACGGAAAGAGTATGA	TGGGCAACCTGGGAGTAG
G6PD	CGAGGCCGTCACCAAGAAC	GTAGTGGTCGATGCGGTAGA
GSS	TACGGCTCACCCAATGCTC	CTATGGCACGCTGGTCAAATA
NQO1	CCTTGTGATATTCCAGTTCCCCC	AACACTCGCTCAAACCAGCC
GCLC	GTTGAGGCCAACATGCGAAA	AGGACAGCCTAATCTGGGAA
GCLM	AGACGGGGAACCTGCTGAA	GATACAATCATGAAGCTCCTCGC

### Western blotting

Cell lysate proteins were resolved on SDS-PAGE and transferred onto a PVDF membrane. Then, the membrane was incubated in a blocking buffer for an hour followed by incubation with primary antibody for 2 h. Primary antibodies for β-actin (#SC-47778) and NRF2 (#sc-365949) were purchased from Santa Cruz Biotechnology, U.S.A.; DPP3 (#PAD907Hu01), G6PD (#PAA716Hu01), GSS (# PAD757Hu01), NQO1 (#PAL969Hu01), and KEAP1 (# PAL648Hu01) were purchase from cloud clone corp, U.S.A. The membrane was then incubated with HRP-conjugated secondary antibody (DAKO, Agilent Technologies, U.S.A.), followed by detection of protein using ECL substrate, and images were taken using a densitometric quantification, which was performed using ImageJ software. Data were normalized first to β-actin and then relative quantification of each protein in knockdown cells was compared with control cells. Experiments were performed in triplicates. On final values, student’s *t*-test was applied using Graphpad prism software.

### Cell proliferation assay/drug sensitivity assay

Cell proliferation was assessed by 3-(4,5-dimethyl-thiazole-2-yl)-2,5-diphenyltetrazolium bromide (MTT) assay. For this, 5 × 10^3^ cells in 100 µl media were seeded in each well of 96-well plates and incubated in a CO_2_ incubator. Then, at various time intervals (0, 24, 48, 72, and 96 h), 100 µl of MTT (2.5 mg/ml) solution was added to each well followed by incubation at 37°C for 3 h in a CO_2_ incubator. Media were removed from each well followed by the addition of DMSO (100 µl/well) to solubilize formazan crystals. Absorbance (O.D.) was recorded at 570 nm using a microplate reader. The O.D. obtained was plotted as a function of time and dose. For the drug sensitivity assay, complete media were prepared using only 1% fetal bovine serum and cells were incubated in different doses of cytotoxic drugs (Cisplatin, 5-Flourourecils, Carboplatin, and Paclitaxel) for 48 h. Absorbance was normalized by the wells with no drugs as 100% viability. Drug response curve were generated using Graphpad Prism software and absolute IC_50_ was calculated. Each experiment was performed in triplicate.

### Colony formation assay

5 × 10^3^ cells were plated in complete media in six-well plates and cultured for 8 days. Media were changed after every 3 days. At the end of 8^th^ day, cells were stained using 0.5% (w/v) crystal violet. Images were taken using digital camera and analyzed by ImageJ2 software to count number and size of colonies along with area of well covered by stained cells.

### Apoptosis assay and cell cycle assay

Cells were seeded in a 25-cm^2^ flask at a density of 0.5 × 10^6^ cells/ml and incubated overnight. Fresh media were added the following day and cells were incubated for 24 h. Following this, cells were harvested for apoptosis and cell cycle assay. For apoptosis assay, cells were stained using Annexin V-FITC/PI Apoptosis Kit (#E-CK-A211, Elabscience) following the manufacturer’s protocol. Briefly, all cells were harvested from the flask and the cell pellet was washed twice with PBS. Cells were then resuspended in 100 μl of 1×Annexin V-binding buffer solution, and 2.5 µl of Annexin V-FITC and 2.5 µl of PI were added to each tube. Cells were vortexed briefly and incubated in dark for 20 min. Following this, the fluorescence was acquired in the flow cytometer (BD FACS Aria, BD Biosciences). The data were analyzed using Kaluza analysis software (Beckman Coulter, U.S.A.). For cell cycle analysis, cells were harvested, washed once with 1X PBS, then fixed in 1 ml of ice-cold 70% ethanol overnight at −20°C. Fixed cells were washed with 1 ml of 1X PBS and were treated with RNAse for 10 min. This was followed by incubation with propidium Iodide (Sigma Aldrich, U.S.A.) for 20 min at room temperature in dark. Following this, cells were analyzed with the flow cytometer. Data were analyzed using Kaluza analysis software (Beckman Coulter, U.S.A.). The percentages of G0/G1, S, and G2/M cells were then calculated using the Michael H. Fox algorithm, which is inbuild into Kaluza software.

### Wound healing assay

5 × 10^5^ Scramble and DPP3-knockdown KYSE-410 and TE-10 cells were cultured into a 24-well plate in RPMI culture medium with 1% FBS. The culture plate was incubated overnight at 37°C in a humidified CO_2_ incubator. After incubation, the media were completely removed and the cell layer was scratched with a sterile 200 μl pipette tip. Cellular debris was removed by washing off with phosphate buffer saline (PBS) two times. The cells were then incubated and imaging was done after developing scratch and at an interval of 24 h up to 72 h. Images of defined scratch positions were taken at different time intervals and the percentage area covered was determined compared with the initial area as 100%. Image analysis was performed using ImageJ2 image analysis software [37]. All samples were plated in quadruplicate.

### Oxidative stress measurement

Scramble and DPP3-knockdown KYSE-410 and TE-10 cells were plated into six-well plates (1 × 10^5^ cells) in RPMI culture medium with 1% FBS. The culture plates were incubated overnight at 37°C in a humidified CO_2_ incubator. After incubation, the media were completely removed and the cell layer was once washed with PBS. After that, 50 mM of 2′,7′-dichlorofluorescein-diacetate (DCF-DA, Sigma #D6883) solution was added to each well and incubated for 20 min. Following incubation, cells were again washed with PBS and cultured in phenol-free RPMI culture medium with 1% FBS. Fluorescence was measured after 4 h of incubation with DCF-DA using flow cytometry.

### RNA-sequencing and data analysis

The TRIzol method was used to isolate RNA. Nanodrop and the Qubit RNA broad-range assay kit were used to measure the amount of RNA using a dye-based qubit spectrophotometer (Thermo Fisher Scientific, U.S.A.). The RNA Integrity Score (RIN) was examined using an Agilent 2100 Bioanalyzer with RNA chips (Agilent Technologies, U.S.A.). For the construction of the RNA-sequencing library, we chose samples having a RIN score of 10. Following the manufacturer’s instructions, a strand-specific Truseq RNA sample preparation kit (Illumina, U.S.A.) was used to prepare samples for sequencing. The Illumina HiSeq2000 equipment was used to sequence 8.0 picomol of the pooled library. Raw data were gathered in the form of ”. fastq” files, with an average of 50 million reads per file. Trimming was carried out using Trimmomatic PE and FastQC v0.11.8 [38] to assess the read quality [39]. We used a Phred grade cutoff 30 filter with a 15-pixel crop up front. The range of reads that continued to be usable after Trimmomatic was >70%. Human reference genome hg38 was obtained from the UCSC genome browser gateway and used for mapping and alignment. Using the splice junction mapper, Tophat2.1.1, reads were aligned to the indexed hg38 genome [40,41]. More than 80% of the total readings was mapped to human genome hg38. The transcripts were assembled and the abundance of the transcripts were estimated. For principle component and pathway analysis, DEG list was uploaded on iDEP (http://bioinformatics.sdstate.edu/idep96) and Genavi web server (https://junkdnalab.shinyapps.io/GENAVi). For differential gene expression and pathway analysis, a cutoff value of log_2_ fold-change of one unit was used. Pathway analysis was performed using default parameters in Genavi web server.

### Statistical analysis

Nonparametric Mann–Whitney test was used for comparing expression in normal/tumor tissues for unpaired samples. For paired samples, Wilcoxon matched pair test was used to compare groups. For cell line studies, all experiments were repeated at least three times and *t*-test was used to compare groups containing repetitive experiments. For pathway analysis, inbuild options were used in iDEP and Genavi webserver. A two-sided *P-*value of 0.05 or lower was regarded as significant. The cBioportal default parameters were used for the survival analysis with a two-sided log-rank test. Graphpad version 8 (Graphstats Technologies Private Limited, India) and STATA software, version 11 (StataCorp LLC, U.S.A.) were used to conduct all analyses.

## Results

### Association of DPP3 expression with clinicopathological features in ESCC

**Figure 1:**
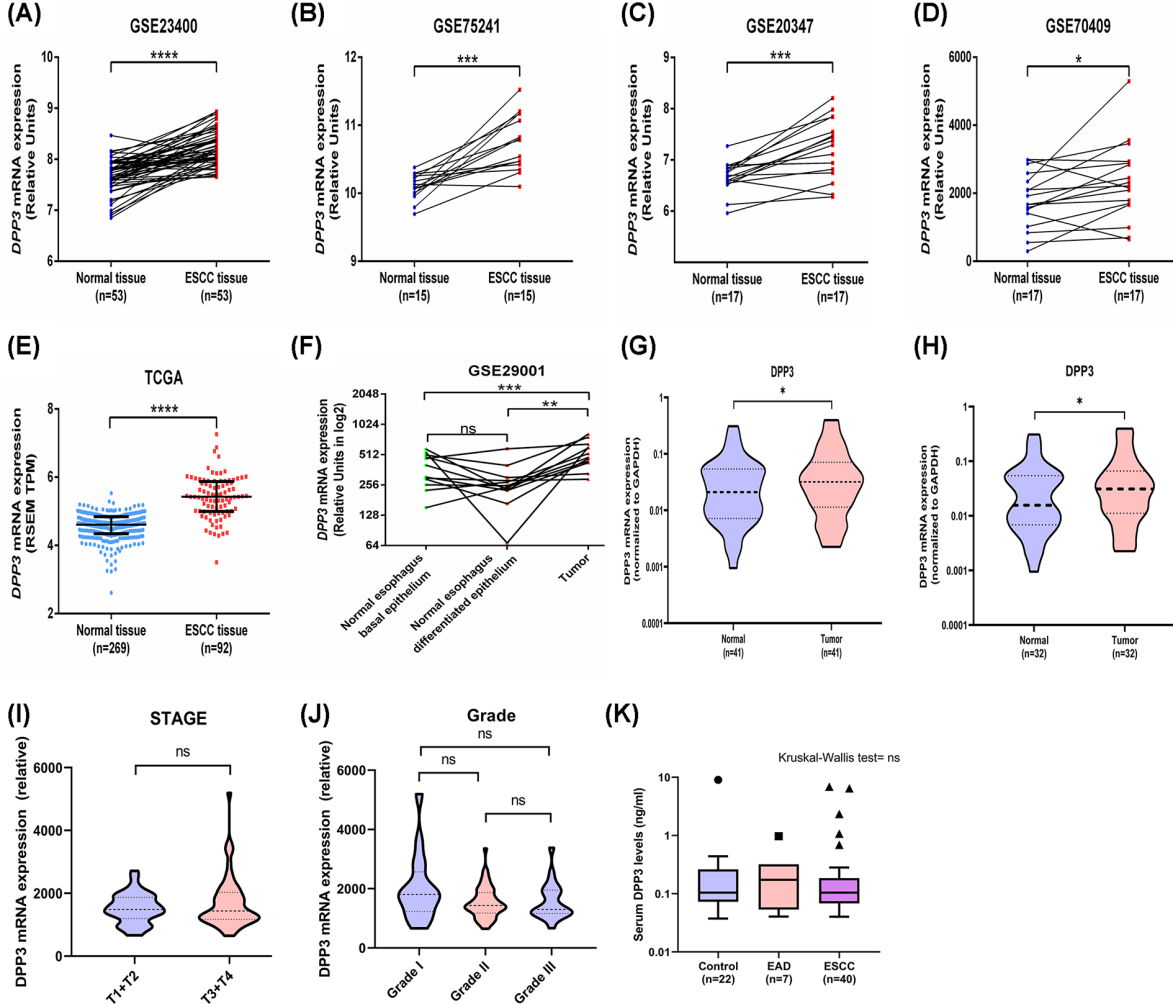
Expression pattern of DPP3 in EC (**A–F**) Comparison of DPP3 mRNA expression between normal esophageal tissue and tumor tissues, (**G**) comparison of DPP3 mRNA expression in normal esophagus and esophageal tumor tissues of institutional cohort, and (**H**) comparison of DPP3 mRNA expression in normal esophagus and ESCC tissues of institutional cohort. (**I**) Association of DPP3 mRNA expression with tumor stage and (**J**) tumor grade in TCGA dataset. (**K**) Comparison of serum DPP3 levels among healthy controls, EAD, and ESCC patients. ns, not significant, *P<0.05, **P<0.01, ***P<0.001, ****P<0.0001.

To determine the expression pattern of DPP3 in ESCC, publicly available RNA-sequencing and gene expression microarray-based datasets of EC were assessed, which suggested that DPP3 exhibits higher expression in tumor tissues compared with paired normal tissues in all the five datasets analyzed (Figure 1A–E). Furthermore, another dataset GSE29001 that utilized microdissected tissues for gene expression profiling also suggested that esophageal tumor tissues exhibit higher expression of DPP3 compared with the normal basal and differentiated epithelium (Figure 1F). This was further confirmed by the assessment of DPP3 mRNA expression in our institutional cohort containing tumor and adjacent normal tissues from 41 EC patients (Figure 1G). Confining the analysis to available 32 ESCC patients of the institutional cohort also suggested higher expression of DPP3 in tumors (Figure 1H). However, a comparison of DPP3 expression based on tumor stage and grade did not suggest any significant difference in DPP3 expression with increasing stage or grade of tumor in the TCGA dataset (Figure 1I,J, respectively). We further compared the DPP3 levels in the serum of ESCC, EAD, and healthy control, which did not suggest any difference among these groups (Figure 1K). Furthermore, serum DPP3 levels were not associated with age, gender, and stage in ESCC patients (Supplementary Figure S1A–C). Additionally, no correlation was observed between serum DPP3 levels and its mRNA expression in the tumors (Supplementary Figure S1D).

**Figure 2:**
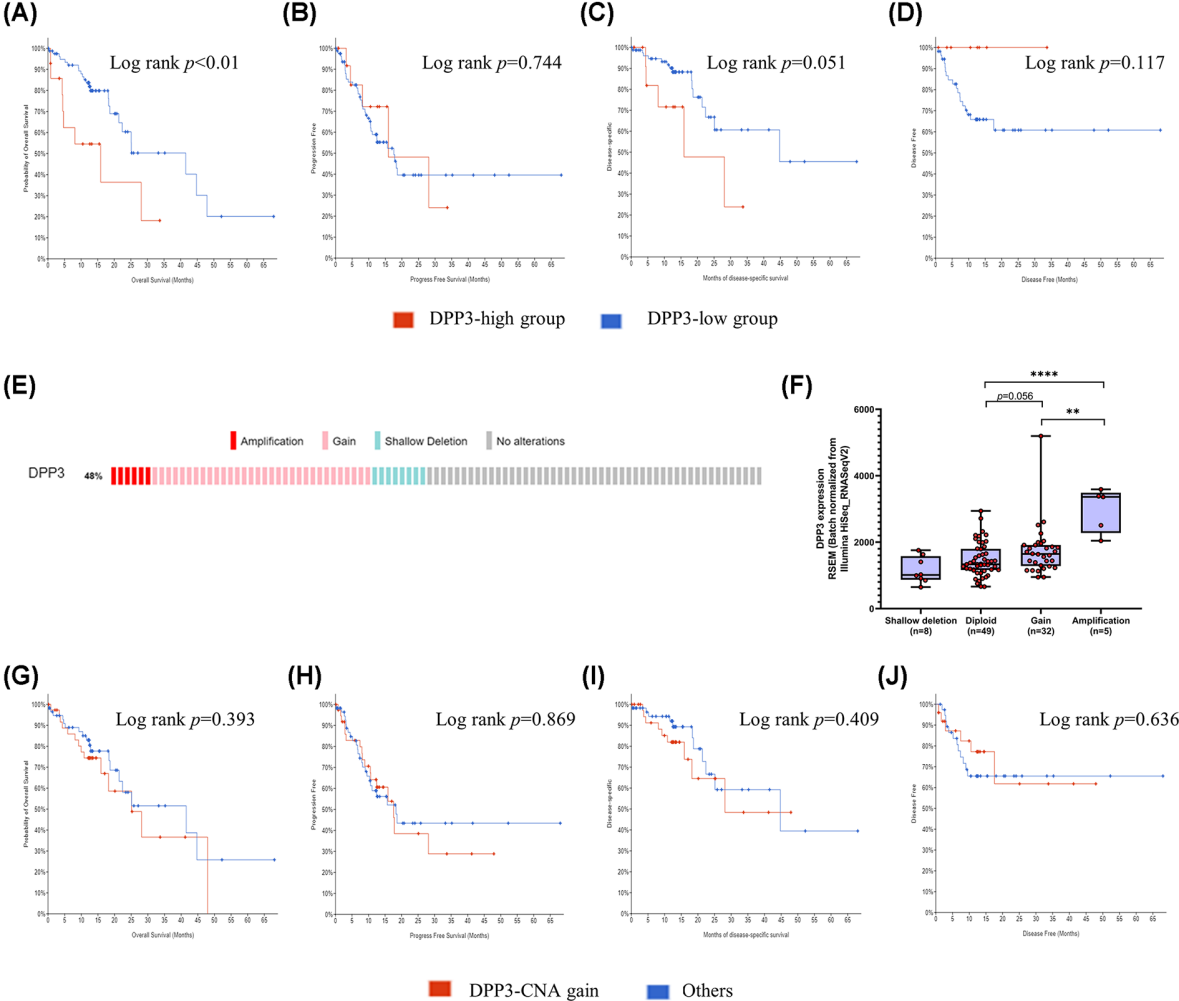
Association of DPP3 with patient prognosis Kaplan–Meier survival analysis for association of DPP3 expression with patient survival in TCGA-ESCC dataset. (**A**) OS, (**B**) PFS, (**C**) DSS, and (**D**) DFS. (**E**) Status of DPP3 copy number in ESCC patients represented as individual columns. (**F**) Comparison of DPP3 expression based on its copy number status among ESCC patients. Kaplan–Meier survival analysis for association of DPP3 copy number amplification with patient survival in TCGA-ESCC dataset. (**G**) OS, (**H**) PFS, (**I**) DSS, and (**J**) DFS. Log-rank *P*-values were retrieved for all survival analyses from cBioPortal. *P<0.05, **P<0.01, ***P<0.001, ****P<0.0001.

Association of DPP3 mRNA expression with patient survival in the TCGA dataset of ESCC revealed that DPP3 high expression group exhibits poor OS (*P*<0.01, Figure 2A) with a median survival of 15.91 months compared with the DPP3 low expression group with a median survival of 41.52 months. Furthermore, association with DSS was observed with a *P*-value near marginal significance (*P*=0.051, Figure 2B) with a median survival of 15.91 vs. 44.74 months in DPP3 high vs. low groups, respectively. There was no difference in survival probabilities of the two groups for PFS (*P*=0.744, Figure 2C) and DFS (*P*=0.117, Figure 2D).

Assessment of DPP3 copy number alteration (CNA) in TCGA-ESCC data (Figure 2E) revealed that among a total of 94 ESCC patients, 8 patients (8.51%) exhibited shallow deletion, 49 patients (52.12%) did not exhibit CNA and labeled diploid, 32 patients (34.04%) exhibited copy number gain and 5 patients (5.31%) exhibited copy number amplification in the DPP3 gene. This suggests that copy number gain in the DPP3 gene is a frequent alteration in ESCC. A comparison of DPP3 mRNA expression based on copy number revealed that its amplification is associated with increased expression in tumors (Figure 2F). However, survival analysis based on DPP3 copy number amplification did not reveal its association with any of the four survival features, i.e., OS, DSS, DFS, and PFS (Figure 2G–J).

### Effect of DPP3 knockdown on cellular processes in ESCC cells

**Figure 3:**
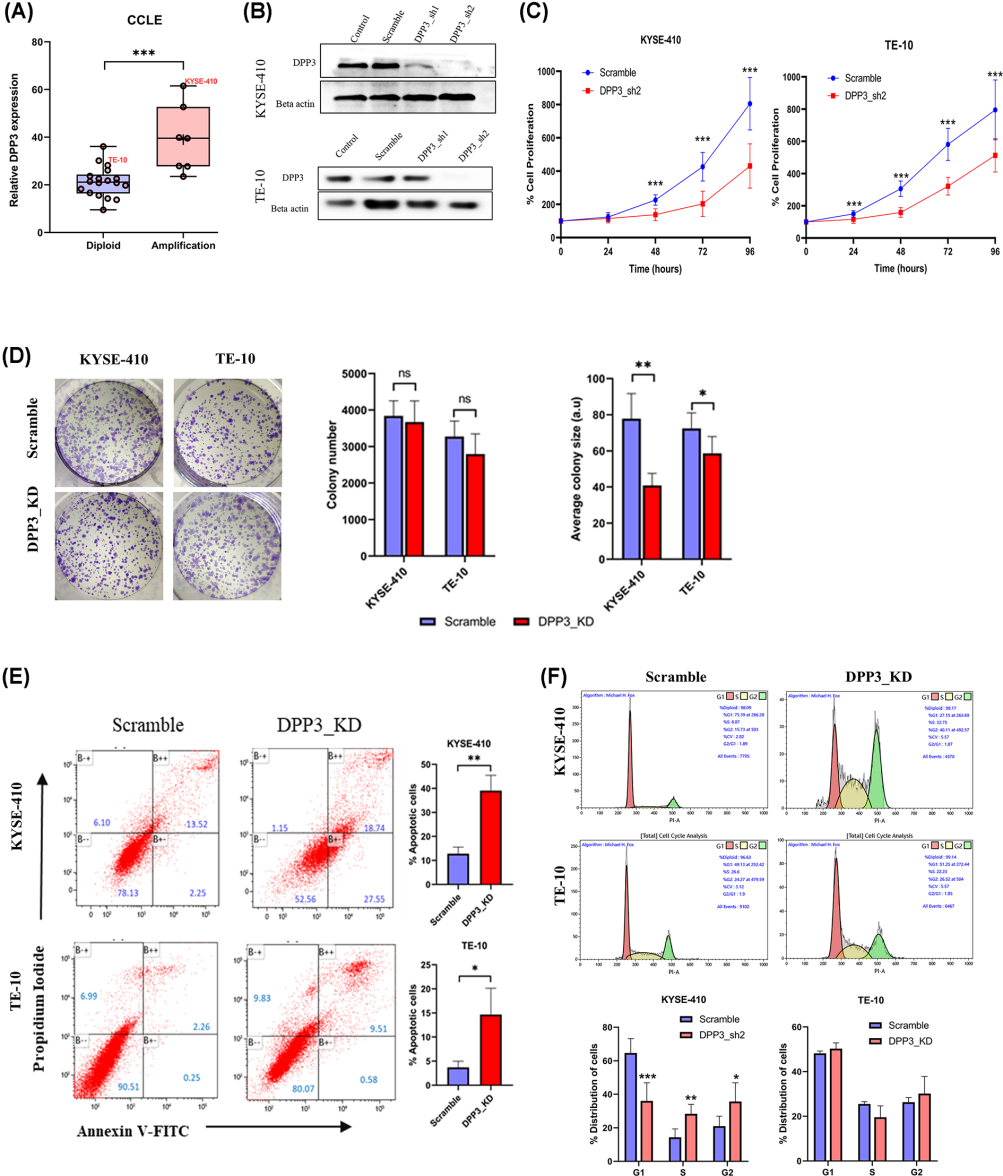
Effect of DPP3 knockdown on cellular functions (**A**) Comparison of DPP3 expression among ESCC cell lines based on copy number status. Data were assessed using cBioPortal web server. (**B**) Immunoblotting for validation of DPP3 knockdown using shRNA. (**C**) MTT assay for effect of DPP3 knockdown on cell proliferation of ESCC cells after DPP3 knockdown. Control and DPP3 knockdown ESCC cells were cultured for different time intervals and MTT assay was perfomed at 0, 24, 48, and 72 h. (**D**) Colony formation assay with comparison of number of colonies formed and average size of colony formed after DPP3 knockdown. (**E**) Apoptosis assay using double staining with Annexin-V and PI to compare rate of apoptosis in control and DPP3 knockdown cells cultured under similar conditions. (**F**) Cell cycle distribution measured by propidium iodide staining. *P<0.05, **P<0.01, ***P<0.001, ****P<0.0001.

**Figure 4:**
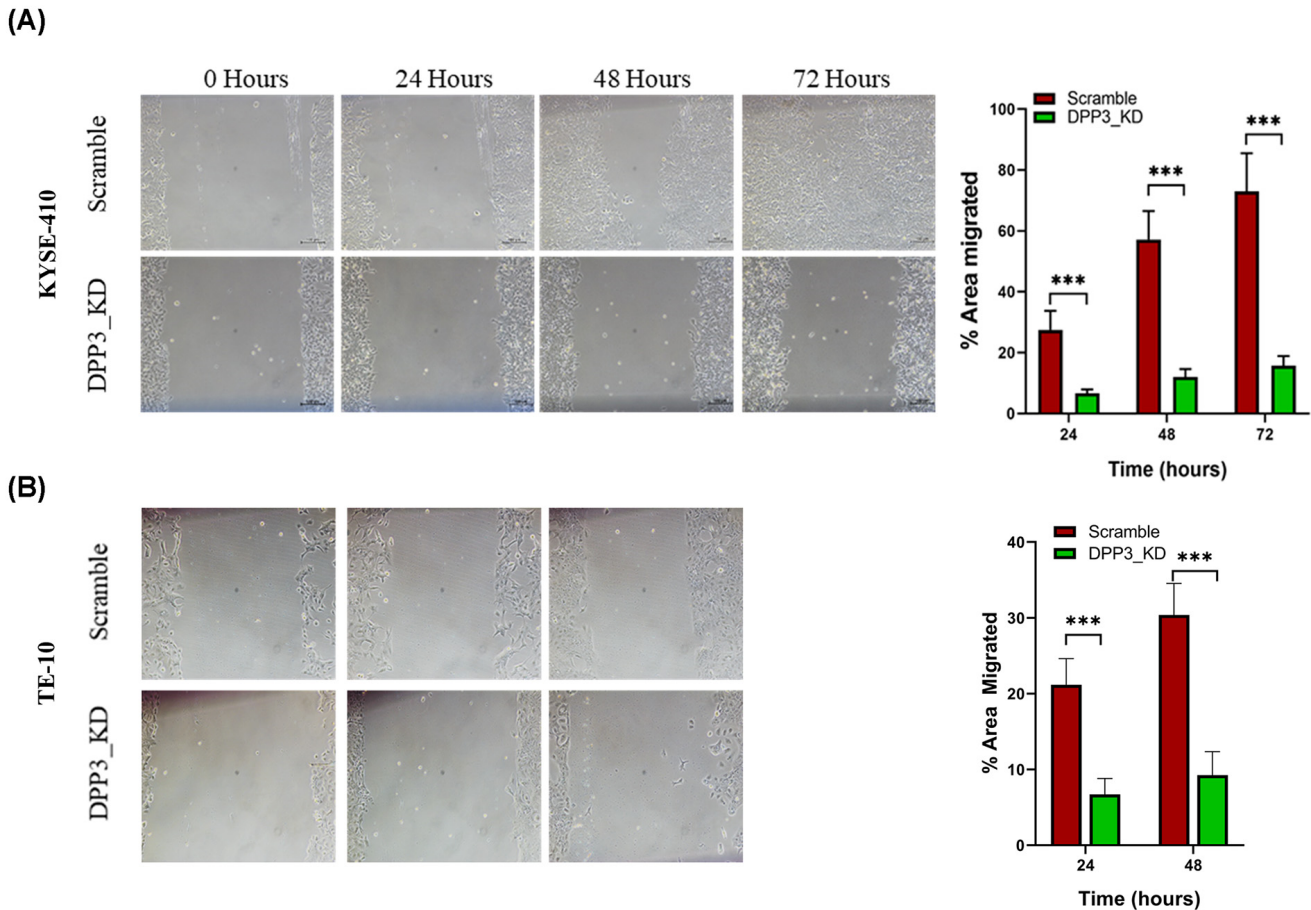
Wound healing assay for effect of DPP3 knockdown on cell migration Representative images show wound healing assay results in control or DPP3 knockdown cells at 0, 24, 48, and 72 h in KYSE-410 and TE-10 cells. *P<0.05, **P<0.01, ***P<0.001, ****P<0.0001.

A gene knockdown approach was utilized to determine the cellular functions of DPP3 in ESCC. To select a suitable ESCC cell line for shRNA-mediated knockdown of DPP3, the mRNA expression and gene copy number alteration data of ESCC cell lines were assessed. It was observed that amongst 25 cell lines assessed for both CNAs and RNA sequencing, seven cell lines (29%) exhibited gene amplification in DPP3 (Figure 3A). Based on relative DPP3 mRNA levels and copy number status, KYSE-410 cells (high DPP3 expression and amplified DPP3) and TE-10 cells (less DPP3 expression and diploid for DPP3) were chosen. Two lentiviral knockdown vectors (DPP3_sh1 and DPP3_sh2) were utilized both of which imparted reduction in DPP3 mRNA and protein compared with scramble control cells (Figure 3B and Supplementary Figure S5). Furthermore, between the two vectors used, cell lines infected with the second vector, labeled DPP3_sh2 (referred to as DPP3_KD hereafter) exhibited higher knockdown efficiency with minimal detection of DPP3 at the protein level. Following DPP3 knockdown, MTT assay was performed at different time intervals revealing significant inhibition of cell proliferation after DPP3 knockdown (Figure 3C). Colony formation assay revealed no effect of DPP3 knockdown on colony-forming ability of cells; however, average colony size was significantly reduced in both KYSE-410 and TE-10 cells (Figure 3D). Furthermore, apoptosis assay revealed that DPP3_KD cells exhibit higher apoptosis compared with control cells in both cell lines (Figure 3E). Assessment of cell cycle analysis revealed that DPP3 knockdown KYSE-410 cells exhibit a reduced fraction of cells in the G0/G1 phase but a higher fraction of cells in both the G2/M and S phases. However, no difference in cell cycle phase distribution was observed in TE-10 cells. To determine the effect of DPP3 knockdown on cell migration, wound healing assay was performed which suggested that DPP3 knockdown significantly inhibited the ability of cells to migrate (Figure 4).

### Effect of DPP3 knockdown on the gene expression profile of ESCC cells

**Figure 5:**
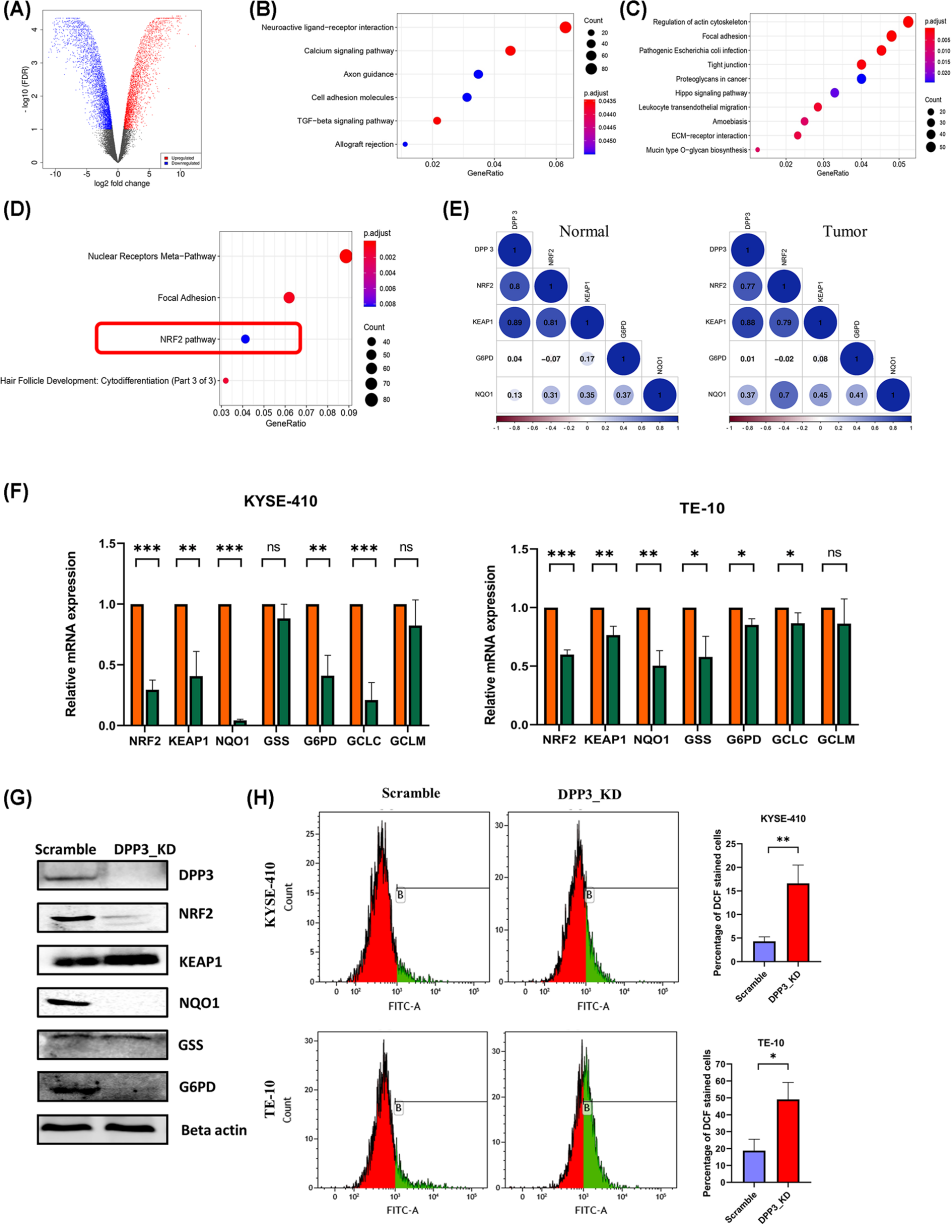
Molecular function of DPP3 in ESCC (**A**) Volcano plot for differential gene expression between control and DPP3 knockdown KYSE-410 cells. (**B**) Up-regulated genes and (**C**) down-regulated genes in KEGG pathway. (**D**) Down-regulated pathways in wikipathway analysis. (**E**) Correlation of DPP3 mRNA expression with expression of NRF2 pathway targets in tumor adjacent normal tissues, and tumor tissues from study cohort. (**F**) Effect of DPP3 knockdown on NRF2 pathway gene’s mRNA expression assessed by qRT-PCR in KYSE-410 and TE-10 cells, and (**G**) validation using immunoblotting in control and DPP3 knockdown KYSE-410 cells. (**H**) Effect of DPP3 knockdown on oxidative stress induction in KYSE-410 cells measured by DCF-DA assay with end point measurement at 4 h in KYSE-410 and TE-10 cells. ns, not significant, *P*<0.05, **P<0.01, ***P<0.001, ****P<0.0001.

Considering the observed effect of DPP3 knockdown on cellular functions such as a reduction in the rate of cell proliferation and increased apoptosis, the gene expression profile of KYSE-410 cells after DPP3 knockdown was assessed (Supplementary Figure S2). Principle component analysis indicated differences in the global gene expression profile between scramble control cells and DPP3_KD cells (Supplementary Figure S2A). A comparison of gene expression profiles suggested 3708 up-regulated and 4422 down-regulated genes in KYSE-410 cells after DPP3 knockdown (Supplementary Figure S2B and Figure 5A).

To determine molecular alterations associated with DPP3, over-representation-based pathway analysis for gene ontology was performed. For biological processes, up-regulated genes in the DPP3 knockdown cells were highly enriched in nervous system-related genes and connective tissues development (Supplementary Figure S3A), while down-regulated genes were enriched in epidermis development and related pathways including wound healing, skin development, and keratinization, cell junction organization (Supplementary Figure S3B). Furthermore, analysis of cellular components in gene ontology revealed significant enrichment of synapsis and related terms, including presynapse, postsynaptic membrane, and ion channel complex among others (Supplementary Figure S3C). In agreement with the observed biological process related to cell adhesion, the down-regulated genes were significantly enriched for cell–cell junction and extracellular matrix-related terms like focal adhesion, apical part of the cell, collagen-containing extracellular matrix, intermediate filament, and desmosome (Supplementary Figure S3D). Similar results were observed for the molecular function analysis, where significant enrichment of ion channels and related terms was observed in overexpressed genes (Supplementary Figure S3E), while down-regulated genes were enriched in cell adhesion, actin binding, integrin binding, ECM binding, and related terms (Supplementary Figure S3F). KEGG pathway analysis for up-regulated genes suggested enrichment of calcium signaling, cell adhesion, TGF-β signaling, and allograft rejection (Figure 5B), while down-regulated genes were enriched in the actin cytoskeleton, focal adhesion, tight junction, leukocyte transendothelial migration, and ECM-receptor interaction (Figure 5C). This suggested that broadly, DPP3 plays important role in cell adhesion and ECM remodeling. Furthermore, pathway analysis was performed based on wikipathways database, which did not show any enrichment for up-regulated genes, while down-regulated genes were enriched in nuclear receptors, focal adhesion, and NRF2 pathway (Figure 5D). This was in agreement with the established role of DPP3 in modulating oxidative stress response; therefore, we further assessed the detailed association of DPP3 with the NRF2 pathway in EC.

### Effect of DPP3 knockdown on NRF2 pathway in ESCC cells

Correlation between mRNA expression of DPP3 and genes involved in the NRF2 pathway was assessed using publicly available gene expression datasets of multiple cancer types in the TCGA study (Supplementary Figure S4). Interestingly, clustering based on Spearman’s correlation value of NRF2 pathway genes revealed that DPP3 exhibited a strong correlation to several NRF2 target genes, which differed widely among tumor types. Furthermore, EC clustered closely with renal clear cell carcinoma followed by breast cancer. Notably, in a previous report on breast cancer, it was shown that NRF2 induction supporting cancer cell survival is enabled by oxidative stress-induced DPP3-KEAP1 interaction [20]. Spearman’s correlation analysis for the mRNA expression of NRF2 pathway genes in normal and tumor tissues from our institutional cohort also revealed high concordance in expression between DPP3 and NRF2 pathway genes, including NFE2L2, KEAP1, and NQO1 (Figure 5E) in tumors, but not in normal tissues. Down-regulation of NRF2 target genes, such as NQO1, G6PD, GCLC, GSS, and GCLM, in DPP3 knockdown cells was also confirmed using qRT-PCR in both KYSE-410 and TE-10 cells (Figure 5F). The difference in expression was most prominent for NQO1 compared with other genes for both the cell lines. Western blot (Figure 5G) further confirmed reduced protein levels of NRF2, NQO1, and G6PD, while expression of GSS was not altered. A comparison of oxidative stress levels in scramble control cells and DPP3 knockdown cells using DCF-DA assay indicated increased levels of oxidative stress after DPP3 knockdown (Figure 5H).

### Effect of DPP3 knockdown on drug sensitivity in ESCC cells

**Figure 6:**
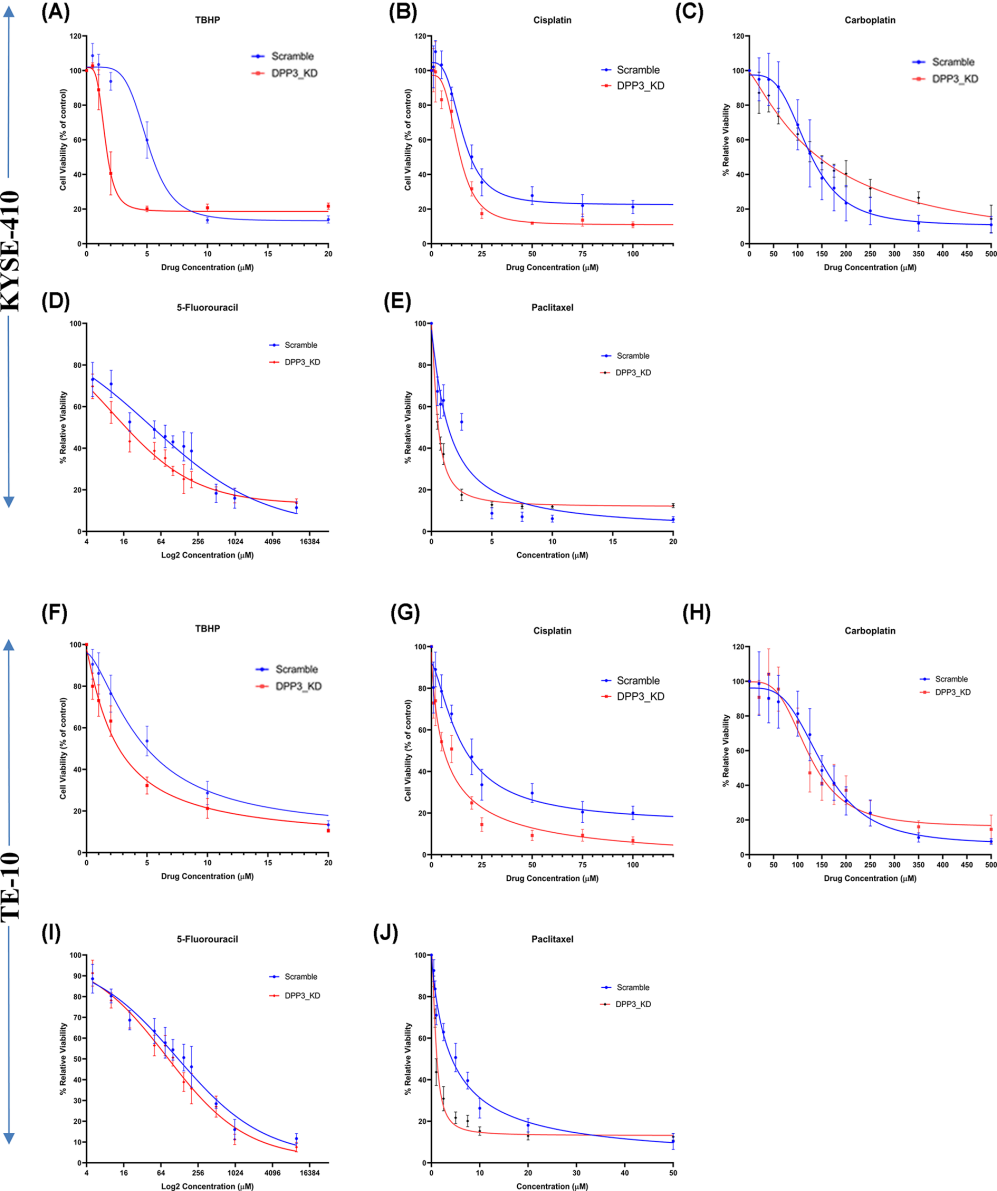
Effect of DPP3 knockdown on oxidative stress and chemotherapy-induced cell death in ESCC cells MTT assay was performed and dose response curve was generated after treating control and DPP3 knockdown cells for 48 h with indicated chemotherapeutic agents, including T-BHP, cisplatin, 5-fluorouracil, paclitaxel, and carboplatin. All experiments were performed thrice and demonstrated figures are representative dose responses in (**A–E**) KYSE-410 and (**F–J**) TE-10 cells by which IC_50_ were calculated.

**Figure 7:**
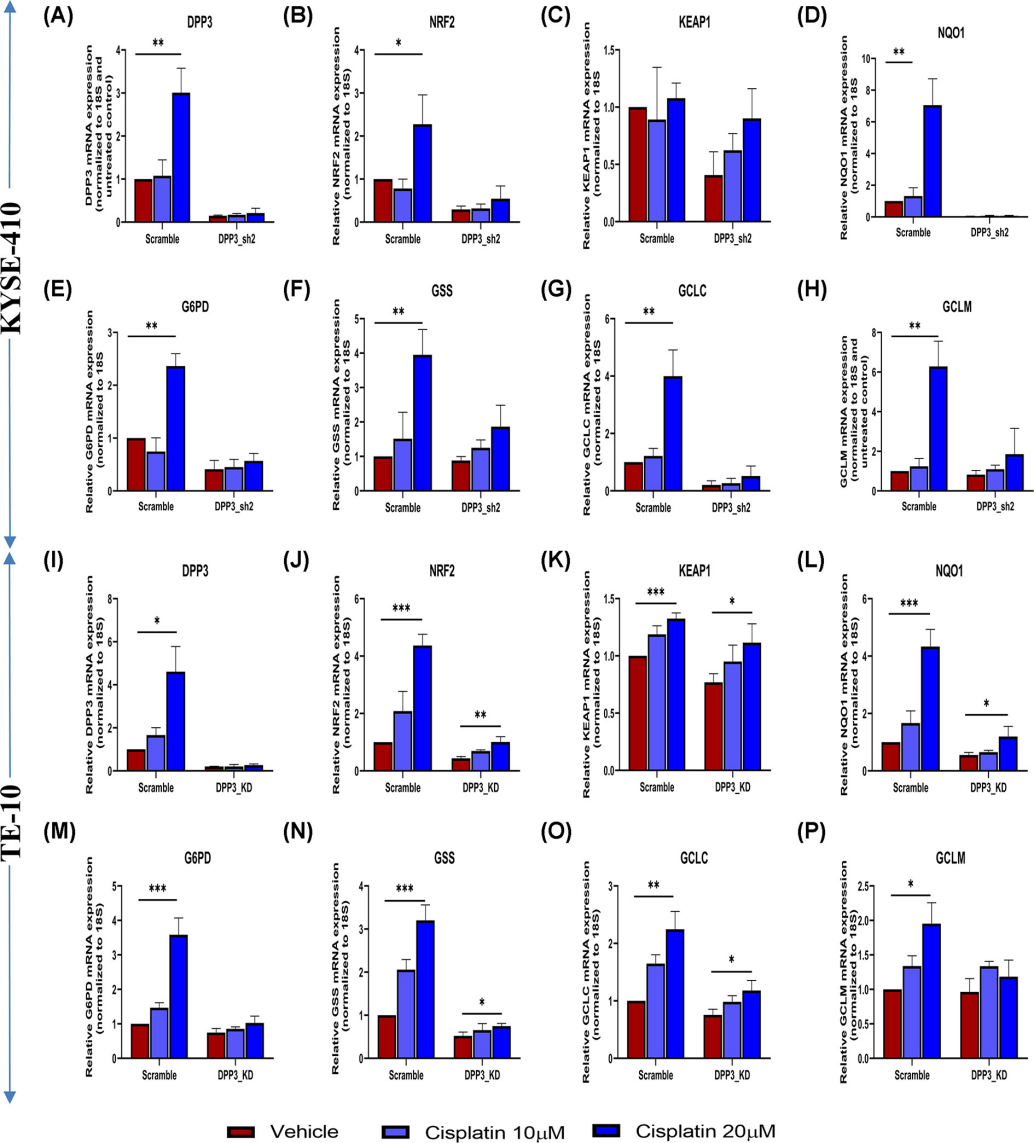
Effect of cisplatin on induction of NRF2 pathway genes in KYSE-410 and TE-10 cells after DPP3 knockdown Scramble control and DPP3 knockdown (DPP3_KD) cells were plated into each well of six-well plates and incubated overnight at 37°C in a humidified CO_2_ incubator. Next day, cells were treated with 10 and 20 µM cisplatin for 48 h. The gene expression of NRF2 pathway genes was normalized by the 2^−ΔCt^ method using 18S rRNA as housekeeping control. Gene expression values of 20 µM cisplatin-treated cells were further compared with respective vehicle (DMSO)-treated scramble control cells using Student’s *t*-test. Figures show bar diagram of relative gene expression of DPP3, and NRF2 pathway genes including NRF2, KEAP1, NQO1, G6PD, GCLC, and GCLM, and GSS after treatment with cisplatin in scramble control and DPP3 knockdown (**A–H**) KYSE-410 and (**I–P**) TE-10 cells. Values presented are mean ± standard deviation of three independent experiments. Results significantly different from untreated cells have been marked with *P<0.05, **P<0.01, ***P<0.001, ****P<0.0001.

To determine the effect of DPP3 knockdown on oxidative stress-induced toxicity in ESCC cells, MTT assay was performed to determine the sensitivity of the ESCC cells to *tert*-butyl hydroperoxide (T-BHP), and chemotherapy (Figure 6 and Table 2). ESCC cells after DPP3 knockdown exhibited higher sensitivity toward T-BHP (Figure 6A,F), cisplatin (Figure 6B,G), and paclitaxel (Figure 6E,J), but not carboplatin (Figure 6C,H). DPP3 knockdown also increased sensitivity of KYSE-410 cells, but not TE-10 cells toward 5-fluorouracil (Figure 6D,I). To determine the effect of chemotherapy in the induction of the NRF2 pathway after DPP3 knockdown, cells were treated with cisplatin, which significantly induced expression of NRF2 pathway genes in control KYSE-410 and TE-10 cells. However, there was limited or no significant induction of NRF2 target genes when cell with DPP3 knockdown were treated with cisplatin (Figure 7).

**Table 2:** Sensitivity of KYSE-410 cells to chemotherapy after DPP3 knockdown. Values given are mean IC_50_ ± SD of three replicated experiments. *P*-value indicates results of Student’s *t*-test

		KYSE-410			TE-10		
S. no.	Drug name/cell group	Scramble	DPP3_KD	*P*-value	Scramble	DPP3_KD	*P*-value
1	T-BHP	5.526 ± 0.439	1.829 ± 0.223	0.0002	5.825 ± 0.4668	3.162 ± 0.3572	0.0014
2	Cisplatin	21.90 ± 2.898	12.58 ± 1.475	0.0077	17.29 ± 2.242	7.850 ± 1.202	0.0030
3	5-Fluorouracil	45.87 ± 7.096	17.60 ± 1.850	0.012	124.7 ± 12.12	105.7 ± 19.41	0.2256
4	Paclitaxel	1.579 ± 0.2973	0.7419 ± 0.2010	0.0156	4.788 ± 0.5692	1.736 ± 0.5925	0.003
5	Carboplatin	114.7 ± 14.94	116.0 ± 15.91	0.9234	172.0 ± 13.31	152.2 ± 13.38	0.1433

## Discussion

DPP family members have emerged as critical regulators of cellular physiology [19]. Several reports on DPP3 have suggested its aberrant activity in cancers of multiple origins, such as breast cancer [20,26,42], lung cancer [24], ovarian cancer [22], endometrial cancer [22], colon cancer [30], and EC [32]. Many of these studies observed higher expression or activity of this peptidase in tumor tissues compared with normal tissues, suggesting its overexpression is common in cancers. While the absence of specific inhibitors of DPP3 limited its utility and research interest, recently its important role in human physiology has surfaced that has again boosted the studies to determine the role of DPP3 in pathophysiology. In the present study, we assessed the potential role of DPP3 in EC. A recent study revealed that DPP3 protein levels are higher in ESCC tissues compared with adjacent normal tissues [32]. Similarly, we assessed the mRNA expression of DPP3 in ESCC patients, which also suggested higher expression of this peptidase in tumor tissues compared with normal esophageal epithelia. The overexpression of DPP3 can be attributed to the gain in its copy number in tumors, as we observed in the present study. This was also reflected in the assessment of DPP3 copy number status in available ESCC cell lines. Similarly, a positive correlation between the genomic copy number of DPP3 and its mRNA expression has been demonstrated in lung squamous cell carcinoma, indicating that gene amplification mediated overexpression of DPP3 is common in malignancies. While circulating DPP3 levels are altered in acute medical conditions [33,34], our assessment of serum DPP3 levels in EC patients does not suggest its alteration in this malignancy. Furthermore, serum DPP3 levels were not associated with tumor stage or age and were not correlated to mRNA expression of DPP3 in tumors.

In context of functional role of DPP3 in ESCC cells, we observed that the down-regulation of DPP3 in ESCC cells led to significant inhibition of cell proliferation suggesting that DPP3 is critical for ESCC cell proliferation. Furthermore, inhibition of cell growth was also evident by the arrest of cells in the G2 and S phases of the cell cycle. Likewise, DPP3 down-regulation led to a higher apoptotic rate in these cells. This agrees with a recent report demonstrating that DPP3 down-regulation is associated with an enhancement in the expression of proapoptotic proteins and alteration in cell cycle distribution in ESCC cells [32]. The reduced proliferation rate and higher apoptotic rate in DPP3 knockdown cells might also be responsible for the observed reduction in colony size of ESCC cells after DPP3 knockdown. Altogether, these results suggested an oncogenic role of DPP3 in ESCC. In agreement to this, DPP3 was shown to be involved in cell cycle regulation and promoting malignant properties in colon cancer cells by involving CDK1 [30]. While our transcriptome analysis in DPP3 knockdown cells did not reveal enrichment of the cell cycle pathway, we did observe an alteration in cell cycle distribution.

Gene ontology analysis suggested significant enrichment of cell–cell adhesion and extracellular matrix-related pathways to be down-regulated after DPP3 knockdown, which may explain our observation that DPP3 knockdown cells exhibit reduced cell migration in wound healing assay. In the context of its observed association with nervous system-related genes, aberrant DPP3 expression has been previously reported in glioma [43]. Most interestingly, the observed down-regulation of the NRF2 pathway as observed in the wikipathway analysis agrees with the existing literature regarding involvement of DPP3 in the activation of the NRF2 pathway. NRF2 signaling is the major pathway regulating the expression of genes in response to oxidative stress and aberrant NRF2 signaling has emerged as a frequent alteration in both esophageal adenocarcinoma and ESCC [12]. Therefore, we performed a detailed assessment of the effect of DPP3 knockdown on NRF2 signaling.

While NRF2 signaling is an essential modulator of the stress response in normal cells, its role in maintaining the structural integrity of normal esophageal mucosa is also well established. Genetic or chemical activation of NRF2 in esophageal mucosa resulted in esophageal hyperproliferation and hyperkeratosis and squamous cell carcinoma in animal models [44]. A previous study determined the NRF2 targets in humans and drosophila and found that NRF2 also regulates the expression of its repressor KEAP1 as a negative feedback loop [45], suggesting that the expression of genes involved in the NRF2 pathway is tightly regulated. In breast cancer, DPP3 was shown to be overexpressed and elevated levels of DPP3 mRNA correlated with increased NRF2 downstream gene expression and poor prognosis [20]. After the role of DPP3 in the regulation of NRF2 pathway activation in breast cancer was described in detail, it was speculated that it might also serve as a therapeutic target in other NRF2-driven malignancies. It was further observed that the involvement of DPP3 in NRF2 pathway induction is independent of its enzymatic activity and the interaction of DPP3 and KEAP1 is enhanced by oxidative stress in breast cancer cells [20]. To note, the authors observed that untreated MCF-7 breast cancer cells exhibited only weak NRF2 levels in the western blot analyses and it significantly increased after around 2 h of H_2_O_2_ treatment. Compared with this, we observed easily detectable levels of NRF2 in KYSE-410 cells at both mRNA and protein levels. While we did not utilize coimmunoprecipitation to demonstrate direct binding of these proteins in the case of EC; however, results demonstrated a significant reduction in the oxidative stress response after DPP3 knockdown in absence of any exogenous oxidative stress, suggesting that this interaction might play a more critical role in ESCC compared with breast cancer. Furthermore, Lu et al. showed that DPP3 depletion did not alter basal levels of NRF2 in breast cancer cells, while we observed it to be reduced in the case of ESCC [20]. One possible explanation of lesser NRF2 levels after DPP3 knockdown could be presence of a positive feedback loop by which, NRF2 signaling regulates its own gene’s (*NFE2L2*) transcription, which has been documented previously [46]. Additionally, DPP3 is primarily involved in proteostasis, and the regulation of proteostasis-related genes by NRF2 is also well-established [47]. We previously reported that an ETS transcription factor-binding motif is critical for the transcriptional regulation of DPP3 in glioblastoma cells [48]. Interestingly, ETS-1 also contains an NRF2-binding site in its promoter region, suggesting a potential mechanism bridging DPP3 and NRF2 [19]. Therefore, these results suggest mutual dependency on DPP3 and NRF2, which may provide novel therapeutic opportunities.

NQO1 is one of the most widely described NRF2 targets so far. In agreement with this, a previous study that first described the role of DPP3 in modulating NRF2 response in neuroblastoma cells also demonstrated NQO1 as a target of this mechanism. Furthermore, G6PD and GCLC also exhibited reduced expression in DPP3 KD cells, suggesting that DPP3 inhibition may highly reduce glutathione synthesis during oxidative stress in esophageal cells. The role of DPP3 in activating the NRF2 pathway was also supported by a recent study, which demonstrated the role of DPP3 in regulating bone homeostasis [49]. It was shown that DPP3 knockout mice exhibit high and sustained oxidative stress and alterations of bone microenvironment leading to bone loss. In line with these results, our results highlighted the role of DPP3 expression in mediating stress response in ESCC, which might also be implicated in other squamous tissues and malignancies associated with aberrant NRF2 activity.

NRF2 pathway is described as one of the major pathways by which eukaryotic cells respond to oxidative stress including cytotoxic chemotherapy. Higher NRF2 pathway activity is associated with drug resistance in cancer cells [11,50]. Therefore, to determine whether the inhibition of NRF2 activity induced by DPP3 knockdown in EC cells directly translated into the alteration of drug sensitivity in these cells, we determined the relative drug sensitivity of the ESCC cells after DPP3 knockdown. In agreement with the observation that DPP3 knockdown leads to reduced NRF2 pathway activation and increased oxidative stress in these cells, we observed that cells attained enhanced chemosensitivity against commonly used cytotoxic drugs, such as cisplatin, 5-fluorouracil, and paclitaxel as well as T-BHP. This also agrees with previous studies [7,11,51]. Furthermore, treatment with the cytotoxic drug cisplatin significantly induced the expression of NRF2 and its targets in control cells, while DPP3 knockdown cells failed to do so, which also confirmed the diminished ability of DPP3 knockdown cells to induce protective response toward chemotherapy-induced oxidative stress.

While the present study provided new insights into the role of DPP3 in malignancies, several limitations remain to be met. The study did not provide information about whether the observed phenotype after DPP3 knockdown was related to a reduction in its protease activity or independent of it. Furthermore, the role of other mechanisms of NRF2 activation, including the role of other disruptor proteins such as p62, PALB2, and SQSTM1 compared with the role of DPP3 was not assessed in the present study and therefore remains to be explored.

## Supplementary Material

Supplementary Figures S1-S5

Supplementary Figures S1-S2

## Data Availability

All data are available from the corresponding author upon reasonable request.

## Abbreviations

CCLE: Cancer Cell Line Encyclopedia
EAD: esophageal adenocarcinoma
ECL: enhanced chemiluminescence
DCF-DA: 2′,7′-dichlorofluorescein-diacetate
DFS: disease-free survival
DPP3: dipeptidyl peptidase III
DSS: disease-specific survival
EC: esophageal cancer
ESCC: esophageal squamous cell carcinoma
GEO: Gene Expression Omnibus
HRP: horseradish peroxidase
MTT: 3-(4,5-dimethyl-thiazole-2-yl)-2,5-diphenyltetrazolium bromide
NRF2: nuclear factor erythroid 2-related factor 2
OS: overall survival
PFS: progression-free survival
PI: propidium iodide
qRT: quantitative real-time
RIN: RNA Integrity Score
T-BHP: *tert*-Butyl hydroperoxide
TCGA: The cancer genome atlas
