# Supplementary material for: Involvement of DPP3 in modulating oncological features and oxidative stress response in esophageal squamous cell carcinoma
Source: Biosci Rep. 2023 Sep 6;43(9):BSR20222472. doi: 10.1042/BSR20222472 (PMC10500228; doi:10.1042/BSR20222472)
Supplement: Supplementary Figures S1-S5 [file BSR-2022-2472_supp.pdf]

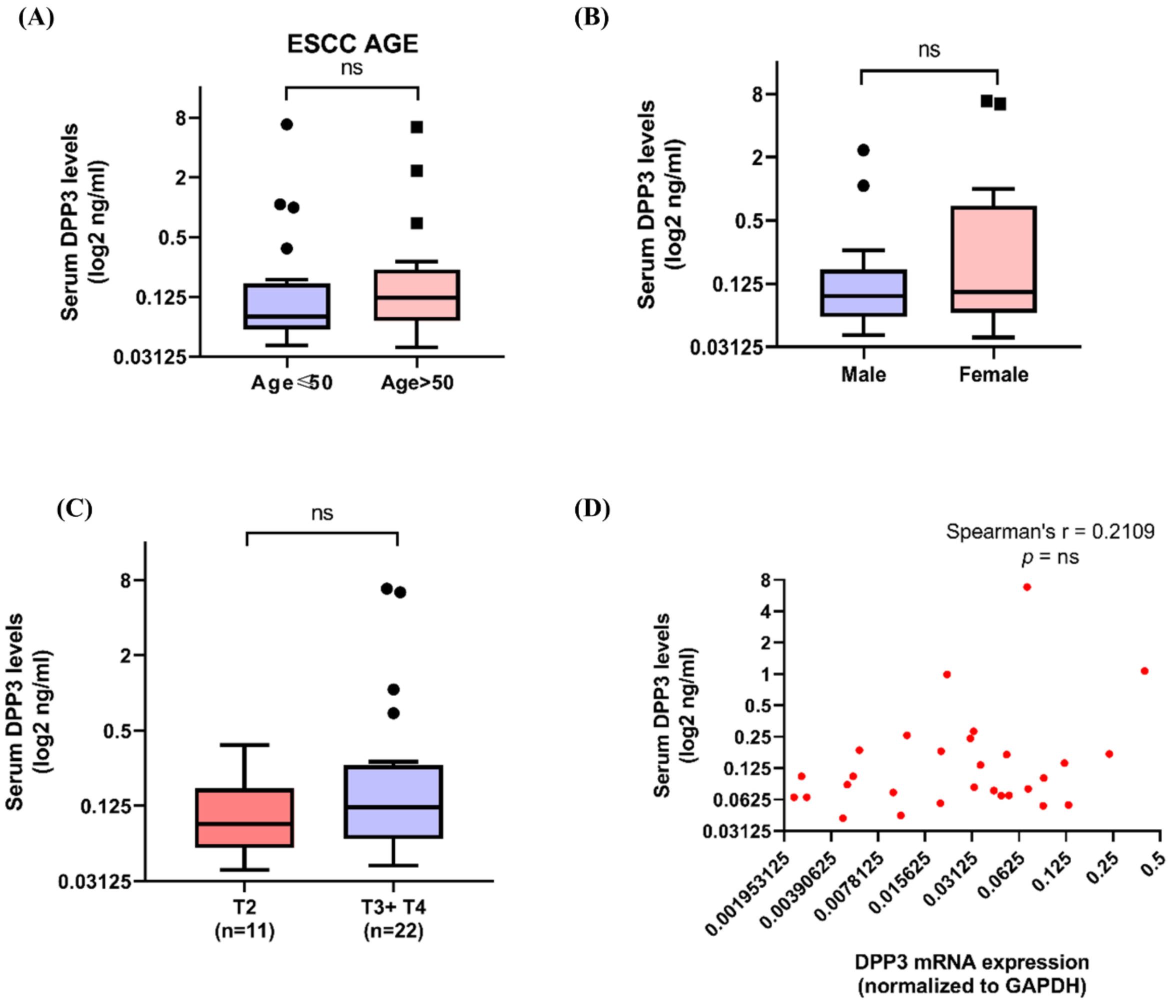

Supplementary Figure S1: Association between DPP3 serum expression with clinicopathological features in ESCC. (A) age, (B) gender, (C) tumor stage. (D) Correlation of DPP3 serum levels with its mRNA expression in tumor tissues of ESCC patients. Groups were compared using Mann-Whitney U test. ns, not significant, * $p<0.05$, ** $p<0.01$, *** $p<0.001$, **** $p<0.0001$.

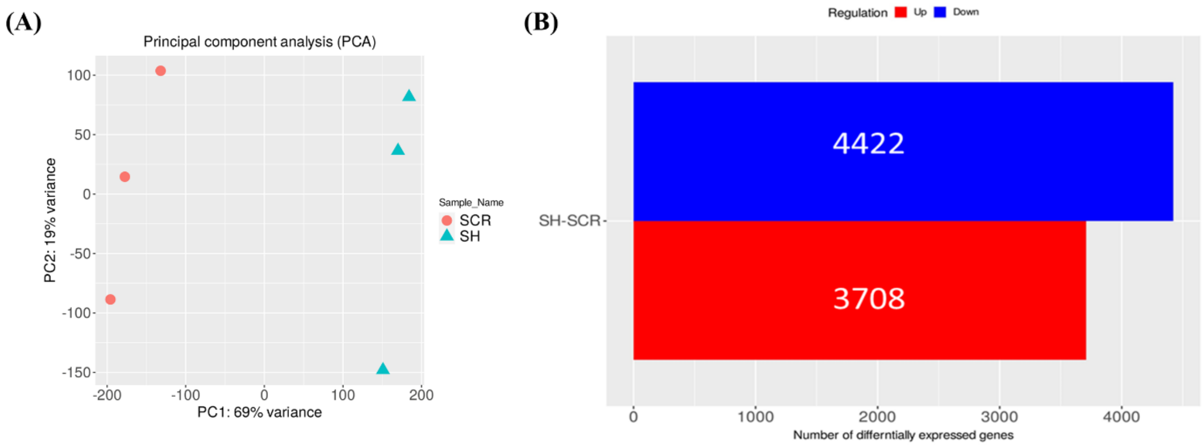

Supplementary Figure S2: Effect of DPP3 knockdown on transcriptome in KYSE-410 cells. (A) Principle component analysis using 1000 most differentially expressed genes, and (B) number of DEG in KYSE_sh2 cells compared to scramble control cells.

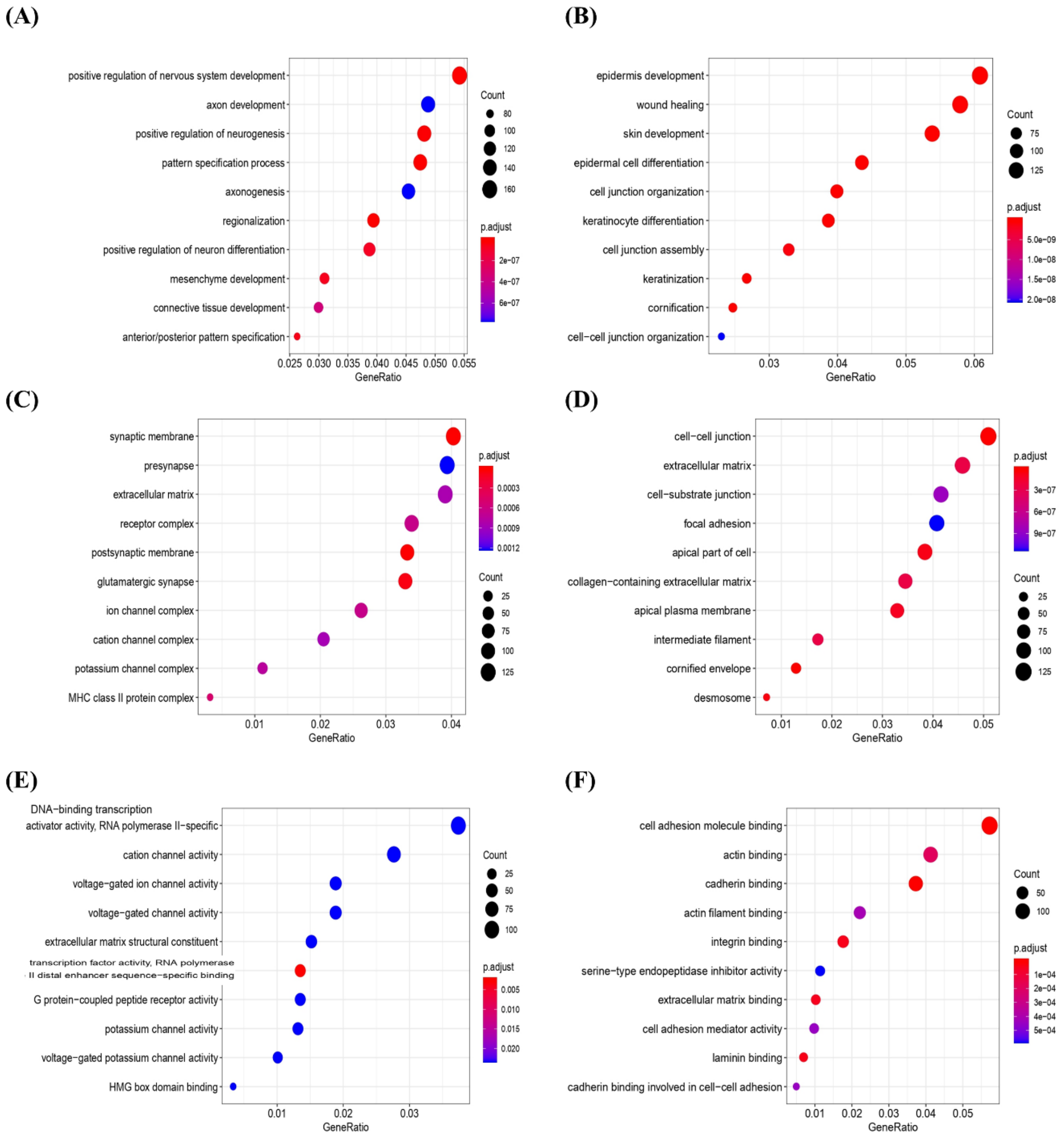

Supplementary Figure S3: Gene ontology analysis after DPP3 knockdown in KYSE-410 cells. (A) Upregulated pathways, and (B) downregulated pathways in biological process. (C) Upregulated pathways, and (B) downregulated pathways in cellular components. (E) Upregulated pathways, and (F) downregulated pathways in molecular functions.

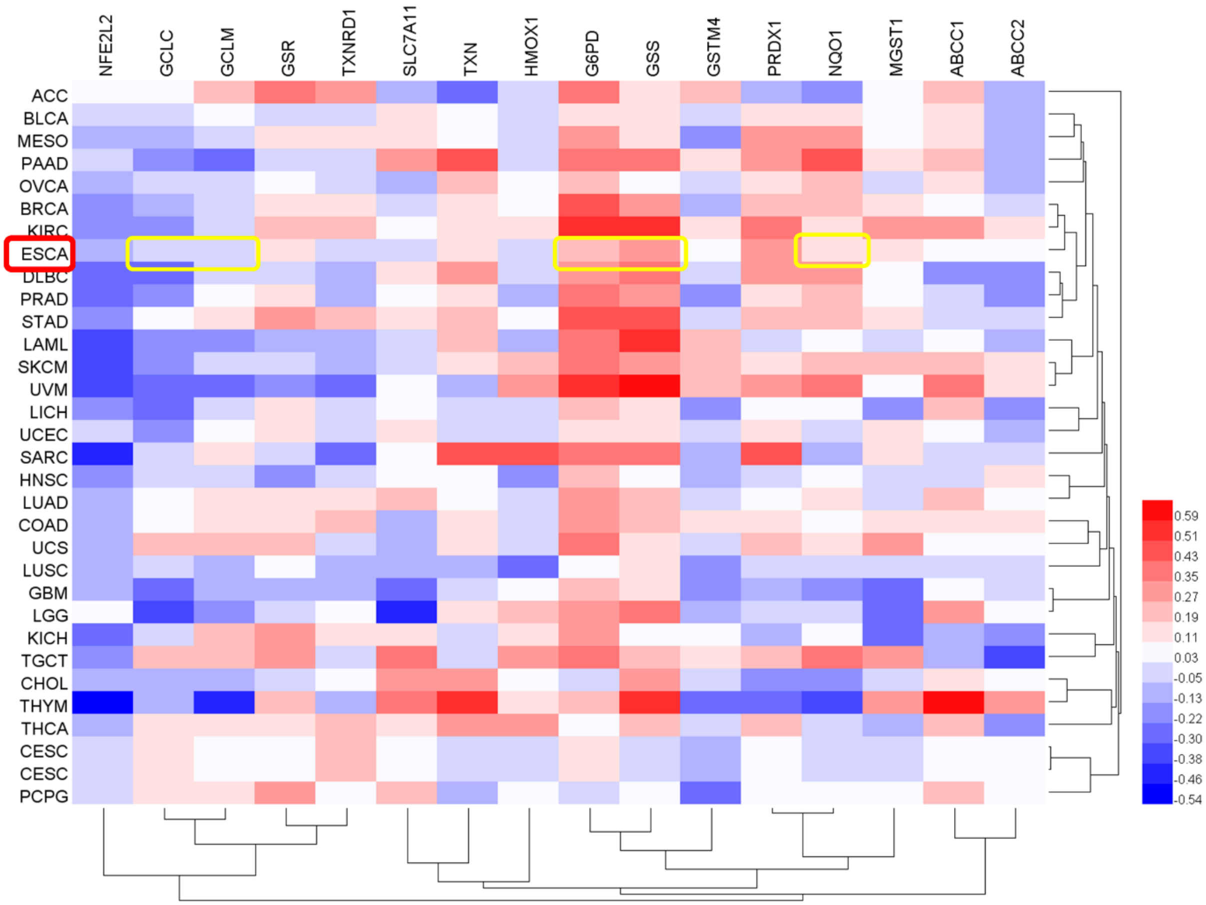

Supplementary Figure S4: Spearman's correlation between mRNA expression of DPP3 and common NRF2 target genes in different cancers from TCGA study. Results of esophageal cancer specific analysis have been highlighted with some genes reported to be altered in cancers. Bladder urothelial carcinoma (BLCA), breast invasive carcinoma (BRCA), cholangiocarcinoma (CHOL), colon adenocarcinoma (COAD), esophageal carcinoma (ESCA), glioblastoma multiforme (GBM), head and neck squamous cell carcinoma (HNSC), kidney chromophobe (KICH), kidney renal clear cell carcinoma (KIRC), kidney renal papillary cell carcinoma (KIRP), liver hepatocellular carcinoma (LIHC), lung adenocarcinoma (LUAD), lung squamous cell carcinoma (LUSC), pancreatic adenocarcinoma (PAAD), pheochromocytoma and paraganglioma (PCPG), prostate adenocarcinoma (PRAD), stomach adenocarcinoma (STAD), thymoma (THYM), and uterine corpus endometrial carcinoma (UCEC), thyroid carcinoma (THCA), ervical squamous cell carcinoma and endocervical adenocarcinoma (CESC), rectum adenocarcinoma (READ), sarcoma (SARC), and skin cutaneous melanoma (SKCM), adrenocortical carcinoma (ACC), lymphoid neoplasm diffuse large B-cell lymphoma (DLBC), acute myeloid leukemia (LAML), brain lower grade glioma (LGG), mesothelioma (MESO), ovarian serous cystadenocarcinoma (OV), testicular germ cell tumors (TGCT), uterine carcinosarcoma (UCS), and uveal melanoma (UVM).

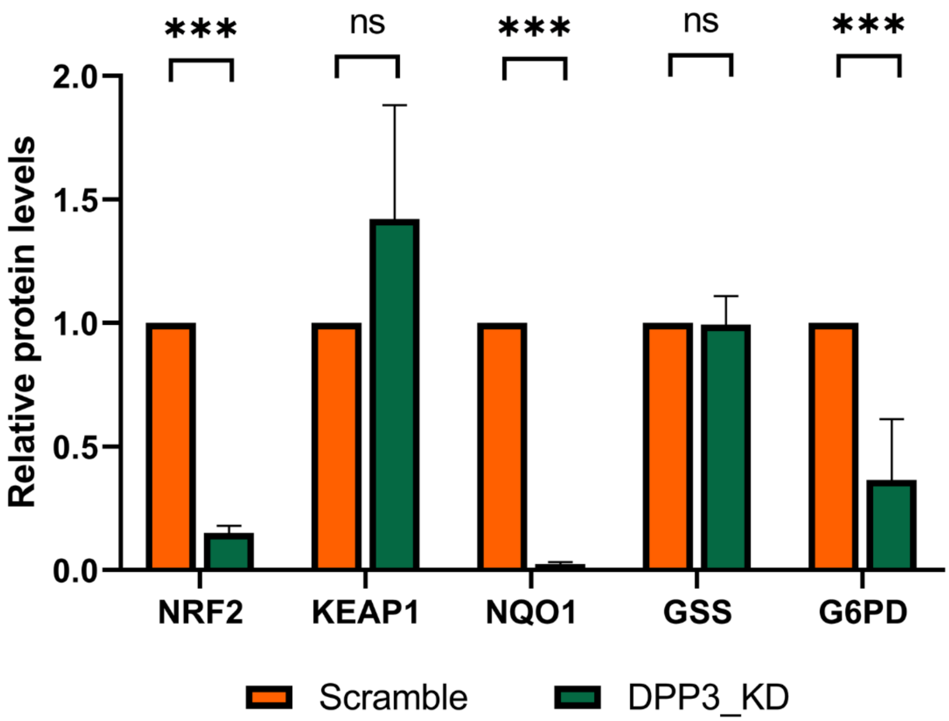

Supplementary Figure S5: Densitometric analysis of proteins levels in western blots shown in figure 5G. The data presents the effect of DPP3 knockdown on protein levels of NRF2 pathway genes. Densitometry quantification was performed on three replicates of western blots for NRF2 pathway proteins. Densitometric quantification was performed using ImageJ software. Data was normalized first to beta actin and then relative quantification of each protein levels in knockdown cells was compared to control cells. On final values, student t test was applied using Graphpad prism software. ns, not significant, $p<0.05$, $^{**}p<0.01$, $^{***}p<0.001$, $^{****}p<0.0001$.
