# Supplementary figures and images for: Involvement of DPP3 in modulating oncological features and oxidative stress response in esophageal squamous cell carcinoma
Source: Biosci Rep. 2023 Sep 6;43(9):BSR20222472. doi: 10.1042/BSR20222472 (PMC10500228; doi:10.1042/BSR20222472)

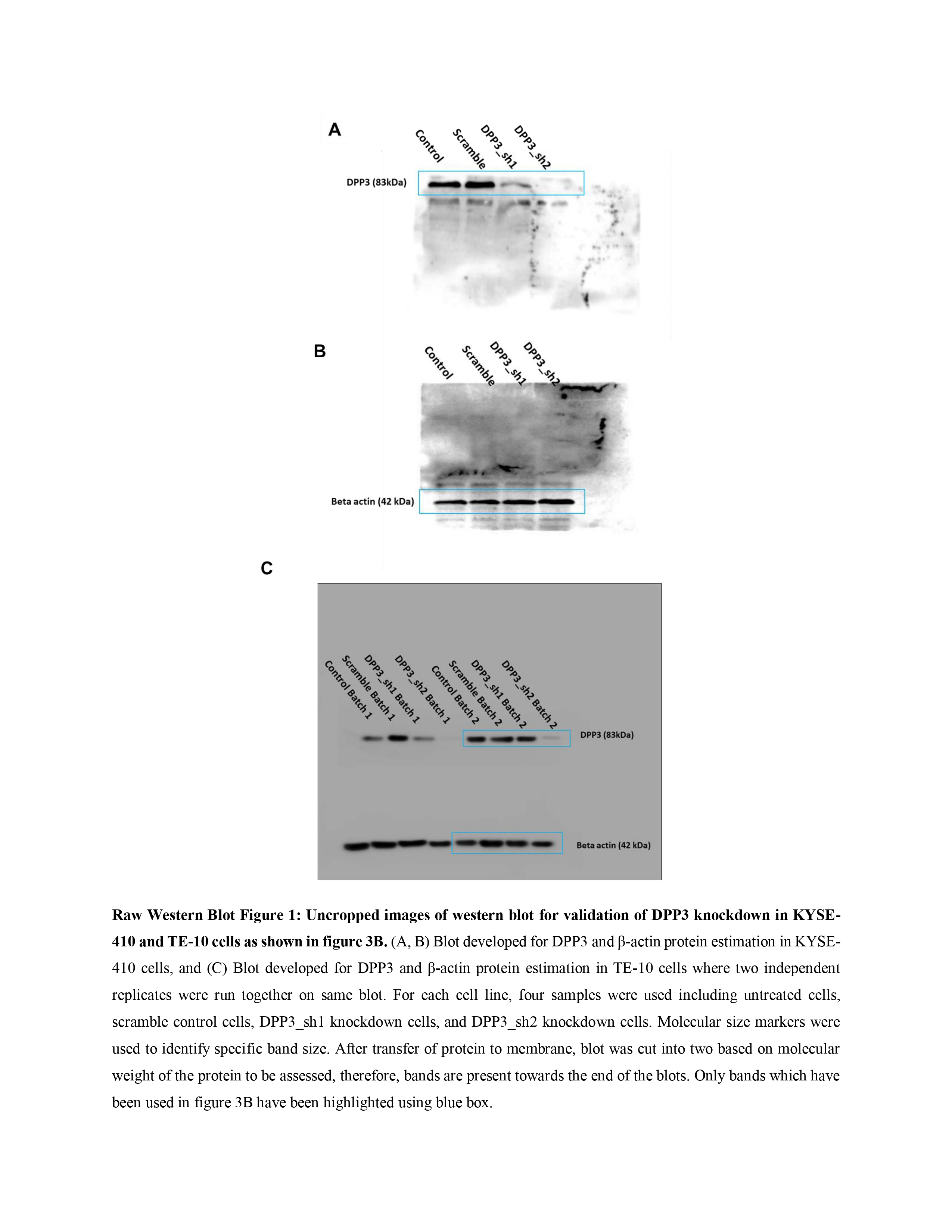

Supplement: Supplementary Figures S1-S2 [file BSR-2022-2472_supp1.zip › BSR-2022-2472_supp1.jpg]

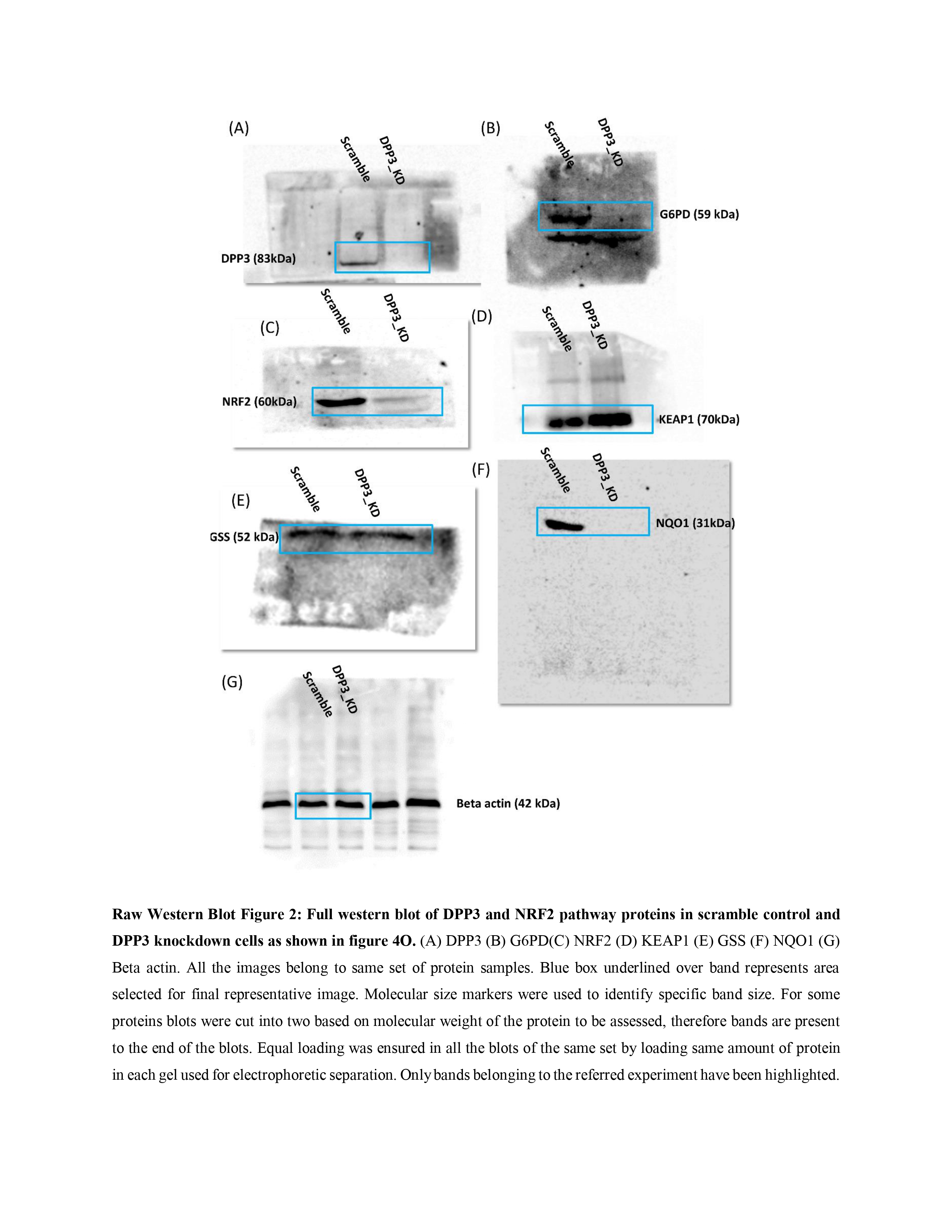

Supplement: Supplementary Figures S1-S2 [file BSR-2022-2472_supp1.zip › BSR-2022-2472_supp2.jpg]
